# Supplementary material for: Detection of air and surface contamination by SARS-CoV-2 in hospital rooms of infected patients
Source: Nat Commun. 2020 May 29;11:2800. doi: 10.1038/s41467-020-16670-2 (PMC7260225; doi:10.1038/s41467-020-16670-2)
Supplement: Supplementary file 1 — Supplementary Information [file 41467_2020_16670_MOESM1_ESM.pdf]

Supplementary Appendix – Chia, et al. Detection of Air and Surface Contamination by SARS-CoV-2 in Hospital Rooms of Infected Patients

Table of Contents:

1. Supplementary Figure 1 - Single general ward airborne infection isolation room layout showing environmental sites sampled and configuration of air samplers.
2. Supplementary Figure 2: Single intensive care unit room layout showing environmental sites sampled
3. Supplementary Figure 3: Cycle threshold values of both clinical samples and environmental samples against the day of illness.
4. Supplementary Table 1: Characteristics of each room and relevant clinical details
5. Supplementary Table 2: All sites sampled, and the number of rooms sampled per site

Supplementary Figure 1: Single general ward airborne infection isolation room layout showing environmental sites sampled (top) and configuration of air samplers (bottom).

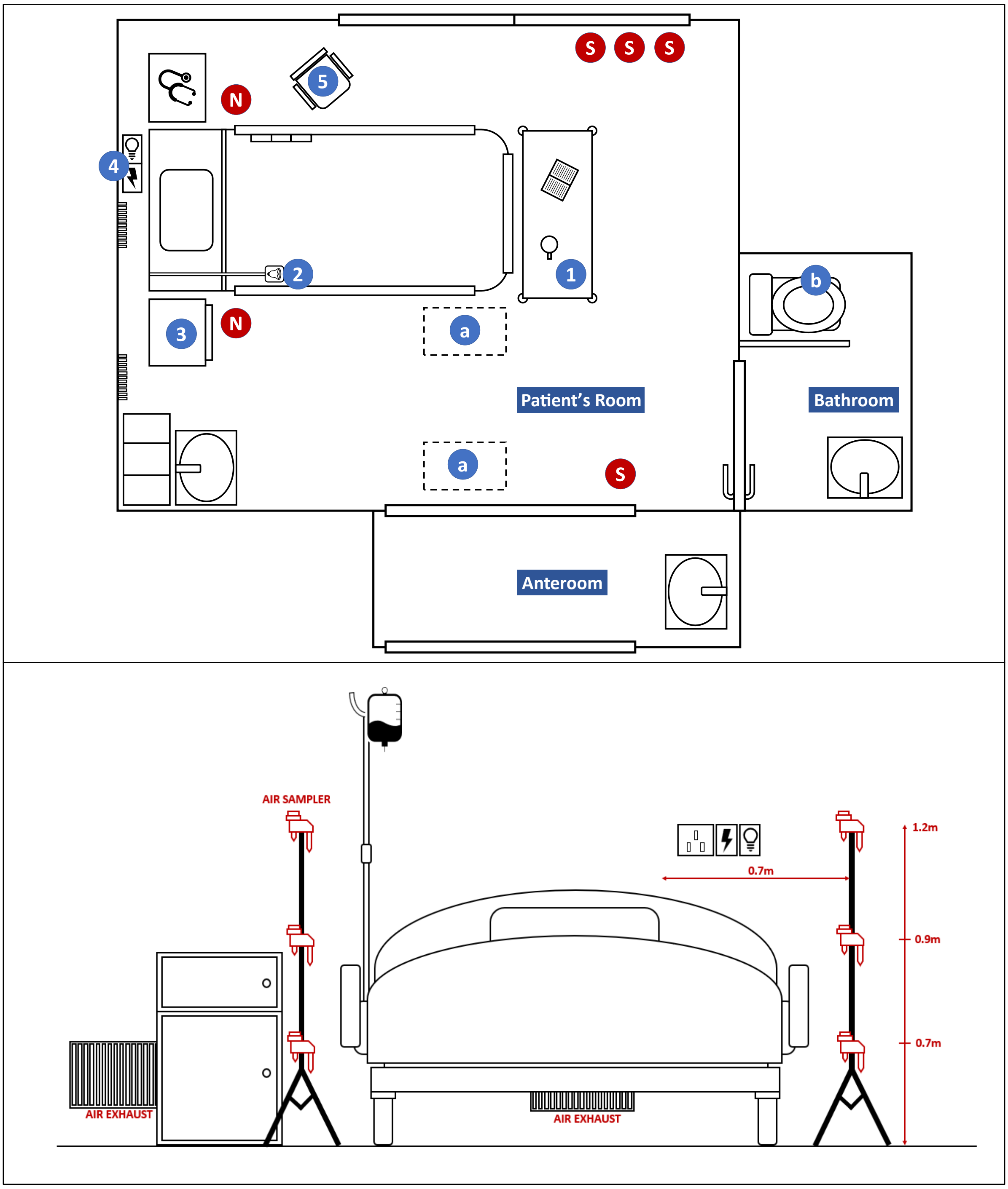

Top: Blue circles mark 5 high-touch areas: (1) cardiac table, (2) bed rail (including call bell), (3) locker, (4) switches, and (5) chair; as well as other key sites sampled: (a) floor, (b) toilet bowl seat. Red circles labelled "N" mark out positions of NIOSH air samplers, while red circles labelled "S" mark out positions of SKC air samplers (only in room 1). During the air sampling duration, patient 1 was seated in the chair while patients 2 and 3 were lying in bed.

Bottom: Configuration of air samplers depicts air sampling layout in rooms 2 and 3 only.

Supplementary Figure 2: Single intensive care unit room layout showing environmental sites sampled

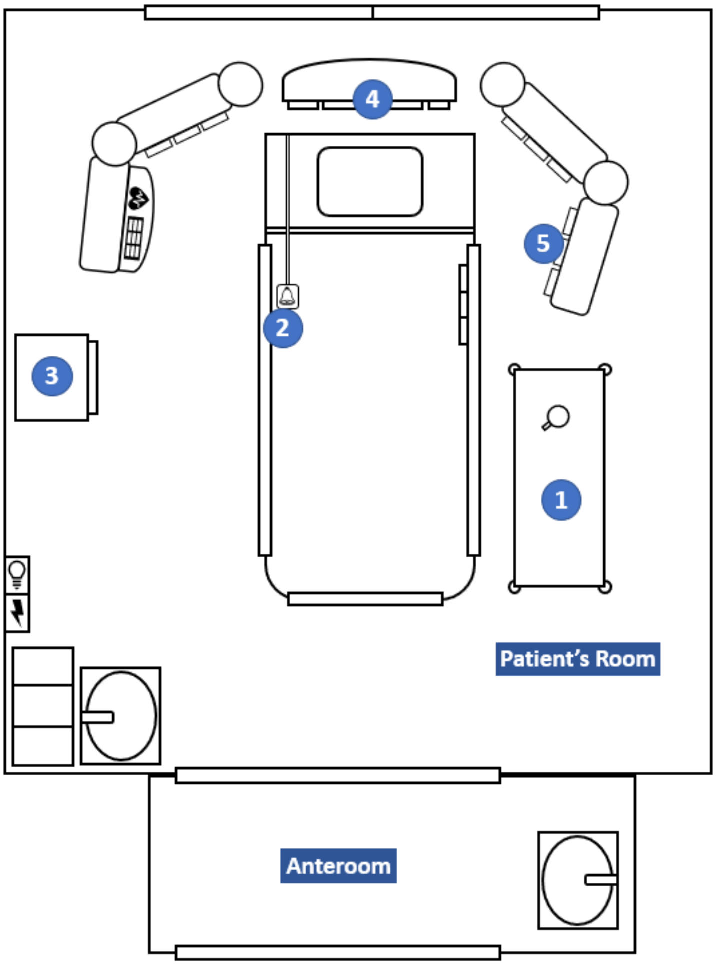

Blue circles mark 5 high-touch areas: (1) cardiac table, (2) bed rail (including call bell), (3) locker, (4) ventilator, and (5) surgical pendants.

Supplementary Figure 3: Cycle threshold values of both clinical samples and environmental samples against the day of illness.

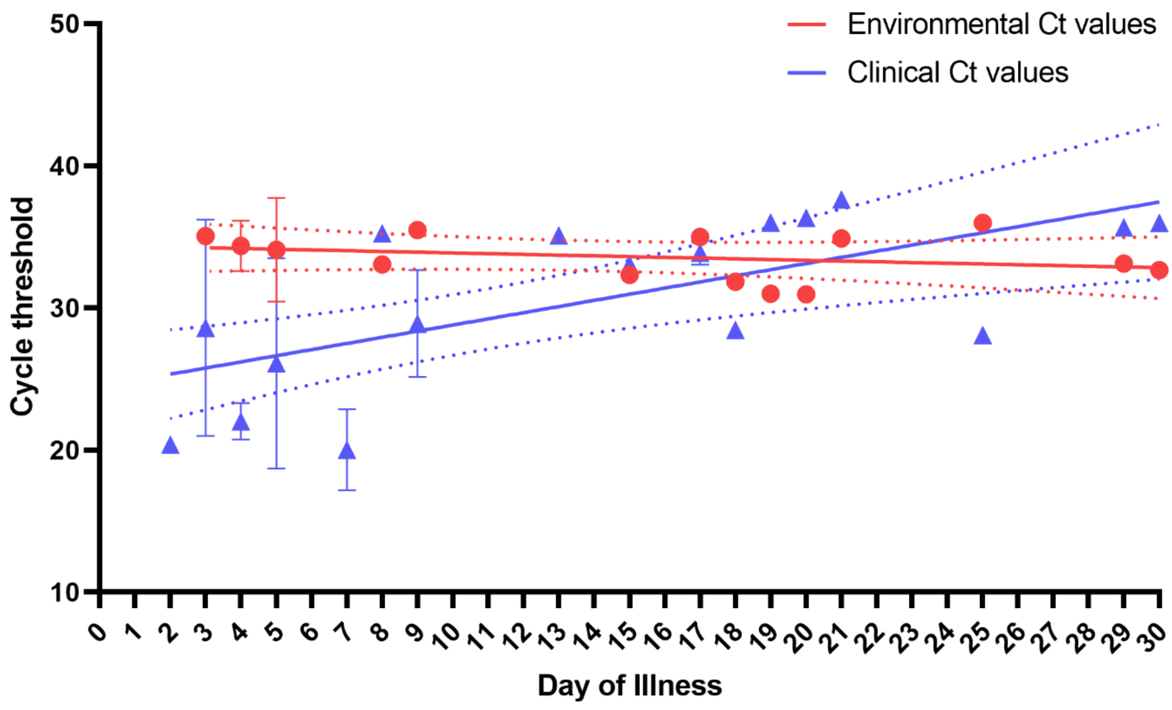

Supplementary Table 1: Characteristics of each room and relevant clinical details

	Clinical Characteristics					Selected Surfaces									Air Sampling		
	**Symptoms on day of sampling**	**Supp. O2 required**	**Day of illness**	**Day of stay**	**Clinical Ct Value**	**Table**	**Bed Rail**	**Locker**	**Switch**	**Chair**	**Ventilator**	**Infusion pumps**	**Floor**	**Toilet**	**Air exhaust vent**	**Air sampling result**	**Particle size fractions**
1	Cough, nausea, and dyspnea	No	9	4	33.22	-	-	-	-	-	Na	Na	-	-	-	-	Not detected
2	Cough and dyspnea	No	5	3	18.45	x	x	x	x	x	Na	Na	x	x	x	x	>4 µm; 1-4 µm
3	Nil	No	5	4	20.11	x	x	-	-	x	Na	Na	-	x	x	x	>4 µm; 1-4 µm
4*	Nausea	Yes	29	20	35.66	-	-	-	Na	Na	-	-	-	Na	Na	Na	Na
5*	Vomiting	Intubated	18	11	28.45	-	-	-	Na	Na	-	-	-	Na	Na	Na	Na
6*	Dyspnea	Yes	5	1	29.48	-	-	-	Na	Na	-	-	-	Na	-	Na	Na
7	Nil	No	30	14	35.98	-	-	-	-	-	Na	Na	x	-	Na	Na	Na
8	Nil	No	25	19	28.09	-	-	-	-	-	Na	Na	-	-	Na	Na	Na
9	Cough	No	21	16	37.63	-	-	-	-	-	Na	Na	-	-	Na	Na	Na
10	Cough	No	20	16	36.34	-	-	-	-	-	Na	Na	-	-	Na	Na	Na
11	Nil	No	17	15	34.48	-	x	-	x	-	Na	Na	-	-	Na	Na	Na
12	Nil	No	17	15	33.28	-	-	x	x	-	Na	Na	-	-	Na	Na	Na
13	Cough	No	19	14	36	-	-	-	-	-	Na	Na	x	-	Na	Na	Na
14	Fever and cough	No	2	1	20.37	-	-	x	-	-	Na	Na	x	-	Na	Na	Na
15	Cough	Yes	15	1	33.04	-	-	-	-	-	Na	Na	-	-	Na	Na	Na
16	Nil	No	13	8	35.09	-	-	-	-	-	Na	Na	-	-	Na	Na	Na
17	Blocked nose and myalgia	No	7	3	22.04	x	x	x	x	x	Na	Na	x	x	Na	Na	Na
18	Cough	No	9	2	26.33	-	-	-	-	-	Na	Na	-	-	Na	Na	Na
19	Fever and sore throat	No	3	2	30.4	-	-	-	-	-	Na	Na	-	-	Na	Na	Na

20	Cough	No	3	3	18.1	x	x	x	-	x	Na	Na	x	-	Na	Na	Na
21	Cough	No	9	3	27.14	-	x	-	-	-	Na	Na	-	-	Na	Na	Na
22	Cough	No	5	3	36.75	x	x	-	-	-	Na	Na	-	-	Na	Na	Na
23	Fever	No	3	2	32.66	-	-	-	-	-	Na	Na	-	-	Na	Na	Na
24	Cough	No	3	2	37.7	-	-	-	-	-	Na	Na	x	-	Na	Na	Na
25	Myalgia	No	3	2	24.16	-	-	-	-	-	Na	Na	-	-	Na	Na	Na
26	Fever, cough, and sore throat	No	4	3	21.1	-	x	x	x	-	Na	Na	-	-	Na	Na	Na
27	Fever, cough, blocked nose, and diarrhea	No	4	3	22.92	x	x	x	-	-	Na	Na	x	-	Na	Na	Na
28	Fever	No	7	3	18.02	-	-	-	-	x	Na	Na	x	x	Na	Na	Na
29	Nil	No	8	8	35.24	-	-	-	-	-	Na	Na	x	-	Na	Na	Na
30	Cough	No	5	3	25.69	x	x	x	x	x	Na	Na	x	x	x	-	Na

x = contamination present
- = no contamination present
Na = not applicable

* ICU rooms. All ICU patients had environmental swabs taken from the ventilator and infusion pumps instead of the electrical switches and toilet. All samples from ICU were all negative.

Supplementary Table 2: All sites sampled, and the number of rooms sampled per site

Environmental Site	No. of rooms sampled
**Patient room**	
Cardiac table	30
Bed rail (including control panel and call bell)	30
Bedside locker	30
Floor	30
Chair	27
Switches over top of beds	27
Air exhaust outlet vent	5
Glass window in the room	5
Ventilator (ICU)	3
Infusion pumps (ICU)	3
Surgical pendants (ICU)	3
Vital sign display screen (ICU)	3
Stethoscope	3
Sink, external surface	3
Sink, internal bowl	3
Interior of room glass door	3
PPE storage area over sink	2
**Toilet**	
Toilet seat & automatic flush button	27
Door handle	2
Hand rail	2
Sink, external surface	2
Sink, internal bowl	2
